# The Characterization of Biodegradable Films and Food Packaging

**DOI:** 10.3390/membranes13100826

**Published:** 2023-10-08

**Authors:** Ismael Marcet

**Affiliations:** Department of Chemical and Environmental Engineering, Research Group TBR, University of Oviedo, 33006 Oviedo, Spain; ismael.marcet@gmail.com

Every year, approximately 300 million tons of petroleum-based plastics is manufactured worldwide, and these plastics cause significant environmental issues due to their non-biodegradable nature and emission of toxic gases upon incineration. Consequently, there is an urgent need to develop new packaging materials that are entirely biodegradable and sourced from renewable substances. Researchers are increasingly focusing on the creation of films and coatings using biodegradable polymers derived from renewable resources, such as lipids, proteins, polysaccharides, microbial polyesters, and polyurethanes. These materials have attracted increasing attention since they can be easily customized, which may provide them with antimicrobial or antioxidant properties, thus making them particularly suitable for food-related applications.

Therefore, the primary objective of this Special Issue was to collate the most significant contributions to the field of biodegradable films for packaging, specifically emphasizing materials with tailored properties designed for specific applications in the food industry.

Thirteen research papers and two reviews were published in this Special Issue. Two research papers focus on the preparation of novel antimicrobial compounds suitable for incorporation into biodegradable food packaging materials. One of these is the article of Valdés et al. [1], which describes the microwave-assisted ethanolic/aqueous extraction of polyphenolic compounds from almond shell residues. The other is a study by Marchianó et al. [2], focusing on the development of a new method for preparing nanovesicles loaded with vanillin, which is a natural compound with antioxidant and antimicrobial properties.

Four other research groups studied the performance of antimicrobial compounds when incorporated into a film matrix. Muñoz-Tébar et al. [3] prepared bioplastics with chia mucilage, which also contained *Origanum vulgare* and *Satureja montana* essential oils, and tested their antifungal, mechanical and physical properties. In addition, Santos et al. [4] prepared PLA-based films with bactericidal activity against *Listeria mono-cytogenes* and *Salmonella enterica* by adding MXene (Ti_3_C_2_T_x_) into the film composition. Furthermore, Utama et al. [5] fermented whey with *Candida tropicalis* and the final product was incorporated into a film formulated with cassava peel starch, which showed antimicrobial activity against *Pseudomonas aeruginosa*. Finally, Hernández-García et al. [6] developed a PLA-PHBV-based film sprayed with ferulic acid, a compound already known to have antimicrobial and antioxidant properties, and then prepared a bilayer film, adding a starch-based monolayer via compression–molding. The resulting bilayer films have stable and long-term antimicrobial and antioxidant properties.

A few famous researchers contributed to this Special Issue with papers related to the preparation of biodegradable packaging materials with improved physical and mechanical properties. This is the case for Carpintero et al. [7], who assessed the plasticizer effect of egg yolk oil on a PLA-based film matrix, which improved the mechanical properties of the membrane and endowed it with antioxidant properties. In addition, Weng et al. [8] investigated the effect of incorporating nanofibrillated cellulose on the mechanical and physical properties of bovine plasma protein-based films. This combination resulted in less-water-soluble films that had higher puncture strength and higher water vapor barrier properties. In this context, Li et al. (2022) [9] grafted halloysite nanotubes and cellulose nanofibers with two silane coupling agents and used these nanomaterials as reinforcement agents to improve the properties of starch-poly(vinyl alcohol) films. Additionally, Shen et al. (2021) [10] assessed the effects of four types of lipids on the water resistance of composite films prepared with wheat bran cellulose/wheat gluten. In this case, using beeswax caused films with higher water resistance and acceptable mechanical properties to be produced.

Another important contribution was made by Amariei et al. [11], who evaluated the possibility of replacing polyethylene-based packaging for foodstuffs with a biodegradable alternative prepared via a mixture of alginate and agar, to which either ascorbic acid or calcium chloride was added. Furthermore, Che Hamzah et al. (2022) [12] extracted anthocyanins from *Brassica oleracea* and used them in the preparation of pH-sensitive sago starch films. According to their results, the films exhibited a noticeable color response at different pHs. The effect of this addition to the starch films was also evaluated. Finally, Sáez-Orviz et al. [13] prepared an edible film with both prebiotic (lactobionic acid) and probiotic (*Lactobacillus plantarum*) ingredients, creating a packaging material with these bioactive properties.

Finally, two reviews are published in this Special Issue. Said et al. [14] cover the performance and characteristics of gelatine-based films prepared from different sources as packaging materials for the food industry. On another note, Mariah et al. [15] studied the present state of research into the utilization of ethylene scavengers in food packaging in order to delay the ripening of fruits and vegetables.

As can be observed, all of these research papers deal with the production of biodegradable packaging materials but focus on very specific areas of interest, such as the production of films with improved antimicrobial properties and films reinforced to be more mechanically flexible or resistant. One paper even explores the nutritional improvement of packaging material. In summary, I am pleased to report that the objectives of this Special Issue have been successfully accomplished. I would like to take this opportunity to express my heartfelt appreciation to all the dedicated authors, meticulous reviewers, and diligent editors who played instrumental roles in ensuring the professionalism and excellence of this Special Issue.
